# Exploring the Threefold Viewpoint on Children’s Oral Health in a Cross-Sectional Study

**DOI:** 10.3390/healthcare12090883

**Published:** 2024-04-24

**Authors:** Anca-Cristina Perpelea, Ruxandra Sfeatcu, Mihail Tușaliu, Mihaela Tănase, Marina Meleșcanu Imre, Alexandra Ripszky Totan, Cristian Funieru, Dragoș Nicolae Nicolescu, Silviu-Mirel Pițuru

**Affiliations:** 1 Department of Organization, Professional Legislation and Management of the Dental Office, Faculty of Dentistry, “Carol Davila” University of Medicine and Pharmacy, 17-23 Plevnei Street, 020021 Bucharest, Romania; dragos-nicolae.nicolescu@drd.umfcd.ro (D.N.N.);; 2 Department of Oral Health and Community Dentistry, Faculty of Dentistry, “Carol Davila” University of Medicine and Pharmacy, 17-21 Calea Plevnei Street, 010221 Bucharest, Romania; 3 Department of Ophthalmology, ENT, Faculty of Medicine, “Carol Davila” University of Medicine and Pharmacy, Eroii Sanitari Boulevard, 050474 Bucharest, Romania; 4 Department of Pedodontics, Faculty of Dentistry, “Carol Davila” University of Medicine and Pharmacy, 17-21 Calea Plevnei Street, 010221 Bucharest, Romania; 5 Department of Prosthodontics, Faculty of Dentistry, “Carol Davila” University of Medicine and Pharmacy, 17-23 Calea Plevnei, 010221 Bucharest, Romania; 6 Department of Biochemistry, Faculty of Dentistry, “Carol Davila” University of Medicine and Pharmacy, 17-23 Plevnei Street, 020021 Bucharest, Romania; 7 Department of Preventive Dentistry, Faculty of Dentistry, “Carol Davila” University of Medicine and Pharmacy, 17-23 Plevnei Street, 020021 Bucharest, Romania

**Keywords:** diagnosis and management, oral health related to quality of life, oral health prevention, public health program, oral habits, healthcare management, legislative prevention

## Abstract

Oral health is situated within the framework of the global health agenda, addressing facets pertaining to well-being and quality of life. The research is based on the need to address variables at the community level to improve schoolchildren’s oral health and promote healthy behaviors and aims to carry out an in-depth analysis from the perspective of the factors that influence children’s oral health. Step 1, designed by the World Health Organization, was utilized. An easy-to-use web interface was created for data collection. The statistical analysis consisted of using multinomial and binominal logistic regression models. The level of education of the adult has a high probability of influencing the consumption of unhealthy or healthy foods, it has a significant probability of exerting influence on social or medical problems and a correlation was found between the level of academic education and the pattern of dental visits. The development of health-promoting behaviors begins in childhood and involves parents, who have an essential role in the education of their children. Oral health promotion programs in schools need to target the child–adult–teacher–dentist relationships. Taking into consideration the aforementioned, a threefold viewpoint is necessary for the development of a national program aimed at promoting the oral health of schoolchildren in Romania.

## 1. Introduction

In 2016, the World Dental Federation (FDI) General Assembly approved a new definition of oral health status [1]. Through this description, oral health is positioned within the global health agenda and addresses aspects related to well-being and quality of life [2]. The basic elements of oral health are “disease and condition status”, “psychosocial function” and “physiological function” [1]. The conceptual framework of oral health created by the FDI is based on the report of the World Health Organization Commission on the social determinants of health. It includes individual, social and environmental factors that influence oral health throughout life [3]. The quality of life related to oral health (OHRQoL) is a complex concept, which covers multiple dimensions and involves biopsychosocial aspects related to the health status of the oral cavity [4].

Socio-economic inequalities have an impact on health at every stage of life, starting from birth [5]. Sanogenic behaviors and those related to the possibility of accessing medical services can be influenced by the social context [6,7]. The main causative factor of dental caries is represented by the consumption of sugar, a fact that highlights a dose–effect relationship [8]. Nutrients play an essential role in maintaining oral health. Food is a factor that contributes to the occurrence of caries, periodontal diseases or other ailments [9]. The link between diet and oral health has been researched and summarized in a variety of articles [10,11,12,13,14] and guidelines have been developed on this topic [15,16,17]. Research has revealed a connection between oral diseases and quality of life [18,19]. The use of oral care services is associated with a variety of obstacles, including educational, health and structural [20]. School is considered an ideal setting for the promotion of positive health and prevention, stimulating awareness of health as the child grows and develops [21,22]. Education plays an essential role in increasing students’ knowledge regarding oral hygiene [22] and attitudes and practices related to healthy behavior.

Currently, in Romania, according to the report by the National Institute of Public Health, there are 467 school dental offices in the urban environment and 1 office in the rural environment [23]. This shows that in order to create an oral health program at the national level, the focus must be on prevention and a collaboration between teachers, dentists, adults and children is necessary, with the common goal of promoting oral health. The study is based on the need to address the association between the perspective on children’s oral health status, attitudes and behavior and the variables at the community level. Thus, the aim is to carry out a detailed analysis from the perspective of the elements that could influence children’s oral health. Their interpretation is based on the perception that the adult has the social perspective, the dietary perspective as well as elements related to his gender and his level of education all relating to the physical impact of the state of the child’s health. 

## 2. Materials and Methods

This study was carried out in the period 2022–2023 in accordance with the subprogram “Evaluation of the oral health status of children and young people”, developed and implemented by the National Institute of Public Health [24]. Using the International Standard Classification of Education (ISCED), students enrolled in public educational institutions in Romania in the ISCED 1 and ISCED 2 educational levels were selected (Table 1) [25,26].

**Figure 1:**
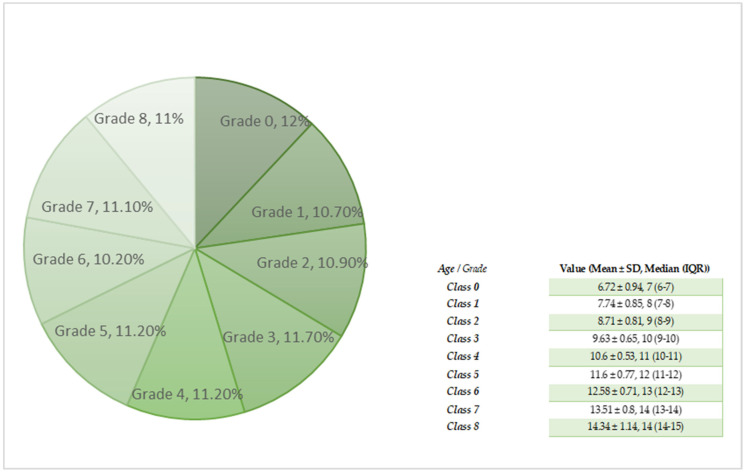
The distribution of study participants according to the class in which they are enrolled in the public education units, corresponding to ISCED 1/ISCED 2 and the mean age.

According to the National Institute of Public Health methodology, students from grade 0 to grade 8 (Figure 1), corresponding to the ISCED primary education (ISCED 1) and lower secondary education (ISCED 2) levels, were selected. Schools with a dental office were selected from all 8 regions according to the Nomenclature of Territorial Units for Statistics of Romania [26].

Before being applied, the Step 1 questionnaire for assessing the state of health and behavior of the children in the opinion of the parent/legal representative was adjusted for the sample taken by the parents of the students and then validated in the Romanian language in a previously published manuscript [26] according to the methodology developed by the World Health Organization in 2020 [26]. The oral health evaluation questionnaire in the parent’s perspective contained questions related to the child’s general information (age, sex, the environment where the child lives and the class the child attends in the public educational institution); information related to the adult’s level of education [26]; questions related to the social impact of the oral health condition (he/she is not satisfied with the appearance of his/her teeth, he/she avoids smiling or laughing because of his/her teeth, other children have fun because of his/her teeth, the toothache or the discomfort caused by this have led to absences from classes); from a medical perspective, questions related to the existence of pain and difficulty during the mastication of hard foods; and information related to eating habits (frequency of consumption of candies, soft drinks, biscuits and fresh fruit).

The questionnaire was self-completed, and an easy-to-use web interface was created for data collection. Thus, errors were minimized. The inclusion criteria for the study were as follows: the existence of an authorized dental clinic within the school, the enrollment of schoolchildren in public educational units in grades 0–8 and the signing of the study participation agreement. Exclusion criteria: the absence of a study participation agreement.

The group of participants included a total number of 3843; the agreement to participate in the study was completed in advance by their legal representative. The distribution related to the class was relatively homogeneous: the most frequent classes in which the children were found were class 0 (12%) and class III (11.7%), where 1790 participants were male and 2053 were female. It was found that 3440 of the analyzed children come from the urban environment, while 403 come from the rural environment [26]. All public education institutions are located in the urban environment. Depending on the place of origin, most of these children (645) come from Bucharest—the capital of Romania [26].

IBM SPSS Statistics 25 was used to perform statistics. Microsoft Office Excel/Word 2021 was used, for example. Testing between groups was performed using Fisher’s Exact Test. The results from the contingency tables were obtained after Z tests with Bonferroni correction. Multinomial and binomial logistic regression models were used to analyze the effect of the level of education, in which univariable models tested the level of education of male and female parents separately (as independent variables), the effect over every tested dependent variable (nominal variables/dichotomic variables), while multivariable models included both levels of education when possible [26]. The performance of the prediction was calculated as odds ratios with 95% confidence intervals along with the significance value (*p*-value). All models were tested for validity of their assumptions, model significance and goodness of fit.

## 3. Results

**Figure 2:**
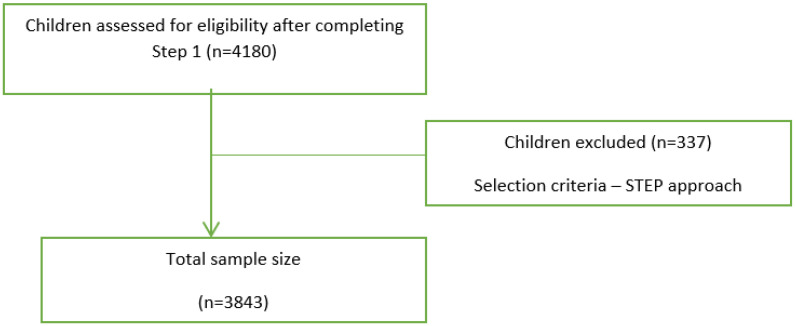
The diagram illustrating the guidelines for selection sample (STROBE Statement).

The study involved 3843 participants [26], enrolled in public schools with authorized dental clinics in Romania (Figure 2). Sample size estimation was made using GPower 3.1.9.7 software. By the design of the study, it was considered that the primary objectives would be the comparison of all analyzed parameters (usually classified as categorical variables with five levels of responses) between education levels (which are four defined levels) in contingency tables using Fisher’s Exact Tests. Therefore, it was estimated that, using a low effect size of w = 0.1 and df = 12, with a minimum power of 0.8 and α = 0.05, the minimum sample size should be 1734 subjects in total. Thus, we consider that selection biases are minimized.

### 3.1. Dietary Perspective

Analyzing the eating behavior of the studied group in the opinion of their parents, the results show the following: most children eat fresh fruit daily (60.6%); biscuits/cakes/pies several times a week (35.6%) or once a day (25.4%); candy several times a month (37.7%), once a week (15.7%) or several times a week (21.6%); drink carbonated or non-carbonated soft drinks several times a month (34.1%), once a week (18.1%) or several times a week (20.9%); eat jam or honey more than one time a month (33.4%) or once a week (20%).

Children who consumed fresh fruit once a day were more frequently associated with female adults who had university education (39.7%) than primary school education (19.4%). Children who consumed biscuits/cakes two or more times a day were more frequently associated with male adults who had primary education (16.7%) than university education (7.7%) and female adults who had secondary school education (17%) than university education (7.3%) (Table 2).

Academic studies of parents decrease children’s chances of consuming sweets and soft drinks, increase the chances of consuming honey/sweets in moderate amounts, decrease the chances of consuming honey/sweets in large amounts daily, decrease the chances of consuming pastries and increase the chances of consuming fresh fruits (Table 3).

Data from Table 3 shows that the existence of academic studies in parents have a significant benefit over children’s food consumption: lowering the odds of very frequent (more than one time/day) candy consumption (for female parents–OR = 0.401, 95% C.I.: 0.233–0.690, *p* = 0.001); lowering the odds of moderate (more than one time/week) soft drink consumption (for female parents–OR = 0.628, 95% C.I.: 0.442–0.891, *p* = 0.009), frequent (one time/day) soft drink consumption (for female parents–OR = 0.346, 95% C.I.: 0.225–0.530, *p* < 0.001) and very frequent (more than one time/day) soft drink consumption (for male parents–OR = 0.505, 95% C.I.: 0.315–0.809, *p* = 0.004 and for female parents–OR = 0.231, 95% C.I.: 0.144–0.371, *p* < 0.001); increasing the odds of very rare (more than one time/month) honey consumption (for female parents–OR = 1.503, 95% C.I.: 1.151–1.963, *p* = 0.003), increasing the odds of having a less frequent pastry consumption from a very frequent level (more than one time/day) to a frequent level (one time/day) (for female parents–OR = 1.656, 95% C.I: 1.147–2.390, *p* = 0.007), to a moderate level (more than one time/week) (for female parents–OR = 1.633, 95% C.I: 1.147–2.324, *p* = 0.006) or to a rare level (one time/week) (for female parents–OR = 1.953, 95% C.I.: 1.322–2.884, *p* = 0.001); and lowering the odds of having a less frequent fresh fruit consumption (thus increasing the overall odds of frequent fresh fruit consumption) from a very frequent level (more than one time/day) to a moderate level (more than one time/week) (for male parents–OR = 0.618, 95% C.I.: 0.480–0.795, *p* < 0.001).

### 3.2. Social Perspective

Regarding the children’s perception in relation to the state of oral health, the results showed that 57.5% of children are satisfied with the appearance of their teeth, whereas 28.6% of children are not satisfied and 502 parents were not aware of their children’s perception.

The observed differences were statistically significant (*p* < 0.001) according to Fisher tests; Z tests with Bonferroni correction show that schoolchildren who were satisfied with the appearance of their teeth were more frequently associated with male adults with university education (72.4%) than those with primary education (52.4%) and female adults with university education (70.9%) than those with high school or gymnasium education (61.9%/49.2%) (Table 4).

Academic studies of adults/parents decrease children’s chances of having social problems (avoiding smiling, having problems with other children, not being satisfied with the appearance of their teeth or missing school due to toothache) (Table 5).

Data from Table 5 show that the existence of academic studies in parents has a significant benefit over children’s social aspects: lowering the odds of avoiding smiling (for male parents–OR = 0.502, 95% C.I.: 0.344–0.733, *p* < 0.001); lowering the odds of having problems with other children (for male parents–OR = 0.428, 95% C.I.:0.188–0.976, *p* = 0.044 and for female parents–OR = 0.422, 95% C.I.: 0.198–0.898, *p* = 0.025); lowering the odds of being unsatisfied with their dental aspect (for male parents–OR = 0.717, 95% C.I.:0.586–0.877, *p* = 0.001 and for female parents–OR = 0.773, 95% C.I.: 0.628–0.951, *p* = 0.015); and lowering the odds of having painful social problems (for female parents–OR = 0.271, 95% C.I.: 0.169–0.436, *p* < 0.001).

### 3.3. Medical Perspective

The data in Table 6 represent the distribution of the participants related to the level of education of the male (M)/female (F) adult and the answer given to the statement “Your son/daughter has difficulties when eating hard foods”. The observed differences were statistically significant (*p* < 0.001) according to Fisher tests and Z tests with Bonferroni correction and highlight that schoolchildren who had difficulties in eating were more frequently associated with male adults who had primary/secondary/high school education (32.1%/24.7%/15.2%) than university education (8.3%); children who had feeding difficulties were more frequently associated with female adults who had primary/secondary/high school education (37.3%/32.1%/16.8%) than university education (9%).

Academic rather than non-academic studies lowers children’s chances of having medical problems (difficulty eating hard foods or chewing food), lowers the frequency of toothaches and lowers the chances of dental pain being the reason for medical consultation (Table 7).

Data from Table 7 shows that the existence of academic studies in parents has a significant benefit over children’s medical aspects: lowering the odds of having tough food difficulty in alimentation (for male parents–OR = 0.655, 95% C.I.: 0.495–0.868, *p* = 0.003 and for female parents–OR = 0.533, 95% C.I.: 0.405–0.701, *p* < 0.001); lowering the odds of having chewing difficulties (for female parents–OR = 0.325, 95% C.I.: 0.168–0.631, *p* = 0.001); lowering the odds of having rare dental pain (for female parents–OR = 0.744, 95% C.I.: 0.596–0.930, *p* = 0.009), occasional dental pain (for female parents–OR = 0.602, 95% C.I.: 0.465–0.781, *p* < 0.001) and frequent dental pain (for female parents–OR = 0.347, 95% C.I.: 0.212–0.566, *p* < 0.001); and lowering the odds of having medical visits for pain treatment instead of regular check-ups (for female parents–OR = 0.242, 95% C.I.: 0.117–0.498, *p* < 0.001).

## 4. Discussion

The development of health-promoting behaviors begins in childhood and involves parents, who have an essential role in the education of their children. It is crucial to evaluate how well children understand the health-promoting message to enhance awareness of their own health condition, foster patient independence and motivation in self-care and bolster their personal autonomy. Taking into account the previously reported results, the level of education of the adult has a high probability of influencing the consumption of unhealthy or healthy foods, it has a significant probability of exerting influence on social or medical problems and a correlation was found between the level of academic education and the pattern of dental visits.

Various research has emphasized the significant influence of social factors on various oral health conditions and behaviors [27,28,29]. Nevertheless, it is noteworthy that the state of oral health relies on the degree to which the individual places value on it [30]. The social impact of the appearance of the oral cavity is proven; there is a clear association between socio-economic factors and the oral health status [18,31]. Children and adolescents whose parents have a higher level of education report a higher daily consumption of fruits and vegetables [22]. The challenge for dentists is to adapt and integrate new models of dental care and general health [32]. Health in all policies (HiAP) is an approach promoted by the World Health Organization in the Ottawa Charter [33] since 1986 [34]. This highlighted the need for an integrated approach to health involving different political fields [35,36]. A fundamental goal of this approach is to reduce inequalities in health [35,37]. HiAP was adopted by the European Union in 2006 [38]. The central point of this approach is that health does not depend only on the medical field, but on several sectors [39]. These policies must be present in every sector. Public health sectors can collaborate with non-health sectors to seek synergies regarding the social determinants of oral and general health [40]. Therefore, the application of health promotion strategies would have a beneficial impact, reducing the prevalence of systemic, but also oral diseases [28].

The role of health policies in shaping health is highlighted in multiple studies [41,42,43]. Public health policies have an essential importance in defining health, focusing on the promotion of well-being, equity and sustainability [33]. Several studies emphasize the need for an integrated approach to health when addressing its social determinants [39,44]. A consolidation of information from multiple sources should contribute to improving the understanding of “health” and in the future, offer new ways to improve health [45]. Globally, this research highlights the need to adopt a complex strategy, which includes the social and environmental factors that influence the state of children’s oral health [46,47,48].

Health education carried out in schools has a beneficial effect on the state of oral health, on children’s knowledge and behavior [49]. The education services offered in schools represent an economical and powerful way of raising standards in the community [21].

The oral health programs conducted in schools must also involve understanding contextual aspects related to the lifestyle and education level of both children and their parents. Consequently, to create an oral health policy, a threefold viewpoint is necessary:

1. Medical perspective: Programs should target the child–dentist relationship in schools. In this educational triad, we have schoolchildren, school dentists and teaching staff. School dentists and teaching staff are the ones who can teach children about health-promoting behavior. They can inform as many children as possible about the necessity of seeking dental care for prevention. Alongside prevention, dentists must also provide curative treatments in school clinics with parental consent. The teaching staff need to be adequately trained to instill healthy habits and practices in children. In Romania, according to the Law nr. 198 of 4 July 2023, article 82, it is mandatory for every pre-university educational institution with legal personality to have a school medical/dental office by 2030 [50].
2. Social perspective: Programs should target child–child and child–adult relationships. Cross-sector collaboration is essential between classes, groups and schools, and in the same geographical areas to promote socialization, communication and relationship-building among children of similar ages. This should incorporate digital interaction to facilitate engagement between children from distant geographic regions, with a specific focus on promoting oral health and understanding intercultural development of social skills.
3. Dietary perspective: There should be informative national campaigns in school regarding the quality and quantity of nutrients that a food provides. Workshops conducted by nutritionists are necessary, with a focus on the characteristics of food and ingredients used, highlighting the benefits and the consequences of consuming different types of food. To be understood by children, this should be approached through play and games. Parents should also be involved, considering their crucial role in their children’s development.

The message must be formulated according to the competence of the subject; for children, an approach is needed that adds specific aspects to the games in order to stimulate the desired behaviors, and for parents, depending on their level of knowledge, there should be signals from the educational (teachers) and medical (school dentists) components.

Oral health promotion programs in schools should target the child–adult–teacher–dentist relationships. Considering the aforementioned, a threefold viewpoint is necessary for the development of a national program aimed at promoting the oral health of schoolchildren in Romania.

Strong points: To the best of our awareness, this represents the first evaluation carried out in Romania that analyzes the three perspectives—medical, social and dietary—in relation to the educational level of the adult according to the questionnaire developed by the World Health Organization in 2013. This study emphasizes the need to develop a prevention strategy that also involves the social determinants of health.

Generalizability: The results can be generalized given the size of the study and the selected age range (5–15 years), which includes the mixed dentition, as well as the adolescent period [51]. Globalization of dental medicine and the need for standardization were respected by using the questionnaire validated in the Romanian language [26,52].

Limitations: the effect of rurality was not analyzed in correlation with the parents’ level of education and the three perspectives: medical, social and dietary; the children studying in schools without authorized dental clinics were not included; the absence of analysis regarding the normative dental treatment need; and inherent biases linked to the data from self-reporting scales, such as bias of social desirability.

Possible future research directions: the correlation of social influence, dietary behavior and medical impact with the state of oral health evaluated by the dentist.

## 5. Conclusions

Present research identifies key components that have a possible influence on the health status of schoolchildren and can constitute a framework for the development of demarcated oral health programs in schools. The results should be used to establish national-level plans in order to reduce social discrepancies and promote good oral health. Thus, the clinicians and researchers were provided with a threefold viewpoint (medical, social and dietary perspectives) for evaluating behaviors related to the educational and dental care needs of schoolchildren. In Romania, there is a need to regulate oral health prevention policies, which also include these visions.

## Figures and Tables

**Table 1:** International Standard Classification of Education–2011, levels of education [25].

International Standard Classification of Education	
ISCED 1	Primary
ISCED 2	Middle school
ISCED 3	High school
ISCED 5–7	Higher education

**Table 2:** Status of food consumption according to parents’ studies.

Candy /Studies–Male	Primary		Middle School		High School		Higher Education		Missing	*p* *
	N.	%	N.	%	N.	%	N.	%		
Never	7	10%	18	13.2%	178	14.8%	227	12.5%	32 (6.93%)	<0.001
More than one time/month	13	18.6%	50	36.8%	441	36.6%	718	39.7%	82 (6.29%)	
One time/week	13	18.6%	18	13.2%	183	15.2%	285	15.8%	46 (8.44%)	
More than one time/week	19	27.1%	22	16.2%	255	21.2%	408	22.5%	43 (5.76%)	
One time/day	7	10%	15	11%	97	8%	126	7%	28 (10.26%)	
More than one time/day	11	15.7%	13	9.6%	51	4.2%	45	2.5%	10 (7.69%)	
Missing	17	19.54%	26	16.05%	89	6.88%	53	2.85%	197 (5.1%) **	623 (16.2%) ***
**Candy** **/Studies–Female**	**Primary**		**Middle School**		**High School**		**Higher Education**		**Missing**	*p* *
	**N.**	**%**	**N.**	**%**	**N.**	**%**	**N.**	**%**		
Never	8	11.8%	18	12.2%	141	14.2%	285	12.9%	10 (2.16%)	<0.001
More than one time/month	14	20.6%	50	34%	347	35%	873	39.6%	20 (1.53%)	
One time/week	12	17.6%	22	15%	161	16.2%	344	15.7%	6 (1.1%)	
More than one time/week	17	25%	25	17%	208	21%	489	22.2%	8 (1.07%)	
One time/day	7	10.3%	17	11.6%	83	8.4%	159	7.2%	7 (2.56%)	
More than one time/day	10	14.7%	15	10.2%	52	5.2%	52	2.4%	1 (0.77%)	
Missing	19	21.84%	29	16.48%	86	7.98%	68	3%	180 (4.6%) **	434 (11.3%) ***
**Soft drinks** **/Studies–Male**	**Primary**		**Middle School**		**High School**		**Higher Education**		**Missing**	*p* *
	**N.**	**%**	**N.**	**%**	**N.**	**%**	**N.**	**%**		
Never	3	4.1%	7	5.2%	119	9.8%	256	14.1%	29 (7%)	<0.001
More than one time/month	11	15.1%	27	20%	359	29.7%	702	38.8%	83 (7.02%)	
One time/week	14	19.2%	16	11.9%	217	17.9%	347	19.2%	32 (5.11%)	
More than one time/week	15	20.5%	39	28.9%	287	23.7%	335	18.5%	48 (6.63%)	
One time/day	14	19.2%	18	13.3%	124	10.3%	112	6.2%	23 (7.9%)	
More than one time/day	16	21.9%	28	20.7%	103	8.5%	58	3.2%	25 (10.87%)	
Missing	14	16.09%	27	16.67%	85	6.57%	52	2.79%	198 (5.1%) **	616 (16%) ***
**Soft drinks** **/Studies–Female**	**Primary**		**Middle School**		**High School**		**Higher Education**		**Missing**	*p* *
	**N.**	**%**	**N.**	**%**	**N.**	**%**	**N.**	**%**		
Never	5	6.8%	9	6.3%	85	8.5%	308	14%	7 (1.69%)	<0.001
More than one time/month	10	13.7%	25	17.4%	279	28%	849	38.6%	19 (1.61%)	
One time/week	14	19.2%	14	9.7%	172	17.3%	420	19.1%	6 (0.96%)	
More than one time/week	14	19.2%	48	33.3%	227	22.8%	424	19.3%	11 (1.52%)	
One time/day	13	17.8%	19	13.2%	125	12.5%	130	5.9%	4 (1.37%)	
More than one time/day	17	23.3%	29	20.1%	109	10.9%	69	3.1%	6 (2.61%)	
Missing	14	16.09%	32	18.18%	81	7.51%	70	3.08%	179 (4.6%) **	429 (11.1%) ***
**Honey** **/Studies–Male**	**Primary**		**Middle School**		**High School**		**Higher Education**		**Missing**	*p* *
	**N.**	**%**	**N.**	**%**	**N.**	**%**	**N.**	**%**		
Never	18	27.7%	37	27.6%	246	20.6%	291	16.1%	55 (8.5%)	<0.001
More than one time/month	20	30.8%	42	31.3%	413	34.6%	601	33.3%	73 (6.35%)	
One time/week	12	18.5%	16	11.9%	238	19.9%	369	20.4%	52 (7.57%)	
More than one time/week	11	16.9%	23	17.2%	201	16.8%	366	20.3%	37 (5.8%)	
One time/day	2	3.1%	12	9%	77	6.4%	163	9%	18 (6.62%)	
More than one time/day	2	3.1%	4	3%	19	1.6%	16	0.9%	5 (10.87%)	
Missing	22	25.29%	28	17.28%	100	7.73%	56	3.01%	198 (5.1%) **	644 (16.7%) ***
**Honey** **/Studies–Female**	**Primary**		**Middle School**		**High School**		**Higher Education**		**Missing**	*p* *
	**N.**	**%**	**N.**	**%**	**N.**	**%**	**N.**	**%**		
Never	11	16.4%	38	27%	221	22.4%	361	16.5%	16 (2.47%)	<0.001
More than one time/month	17	25.4%	41	29.1%	311	31.6%	765	34.9%	15 (1.31%)	
One time/week	15	22.4%	18	12.8%	211	21.4%	432	19.7%	11 (1.6%)	
More than one time/week	13	19.4%	31	22%	157	16%	431	19.6%	6 (0.94%)	
One time/day	5	7.4%	10	7%	65	6.6%	186	8.5%	6 (2.21%)	
More than one time/day	6	9%	3	2.1%	20	2%	17	0.8%	0 (0%)	
Missing	20	23%	35	19.89%	93	8.63%	78	3.44%	178 (4.6%) **	458 (11.9%) ***
**Pastries** **/Studies–Male**	**Primary**		**Middle School**		**High School**		**Higher Education**		**Missing**	*p* *
	**N.**	**%**	**N.**	**%**	**N.**	**%**	**N.**	**%**		
Never	3	4.1%	1	0.7%	25	2%	32	1.8%	3 (4.69%)	<0.001
More than one time/month	10	13.9%	19	13.5%	153	12.4%	199	10.9%	39 (9.29%)	
One time/week	4	5.6%	16	11.3%	228	18.5%	291	15.9%	40 (6.91%)	
More than one time/week	34	47.2%	56	39.7%	408	33.2%	678	37.2%	72 (5.77%)	
One time/day	9	12.5%	30	21.3%	310	25.2%	484	26.5%	59 (6.61%)	
More than one time/day	12	16.7%	19	13.5%	106	8.7%	141	7.7%	26 (8.55%)	
Missing	15	17.24%	21	12.96%	64	4.95%	37	2%	199 (5.1%) **	575 (14.9%) ***
**Pastries** **/Studies–Female**	**Primary**		**Middle School**		**High School**		**Higher Education**		**Missing**	*p* *
	**N.**	**%**	**N.**	**%**	**N.**	**%**	**N.**	**%**		
Never	3	4.2%	3	1.9%	19	1.8%	36	1.6%	3 (4.69%)	<0.001
More than one time/month	9	12.7%	24	15.7%	130	12.9%	249	11.2%	8 (1.9%)	
One time/week	5	7%	24	15.7%	168	16.7%	376	17%	6 (1.04%)	
More than one time/week	33	46.5%	39	25.5%	346	34.3%	313	36.7%	17 (1.36%)	
One time/day	10	14.1%	37	24.2%	250	24.8%	581	26.2%	14 (1.57%)	
More than one time/day	11	15.5%	26	17%	96	9.5%	163	7.3%	8 (2.63%)	
Missing	16	18.39%	23	13.07%	69	6.4%	52	2.29%	176 (4.5%) **	392 (10.2%) ***
**Fresh fruit** **/Studies–Male**	**Primary**		**Middle School**		**High School**		**Higher Education**		**Missing**	*p* *
	**N.**	**%**	**N.**	**%**	**N.**	**%**	**N.**	**%**		
Never	3	4.1%	2	1.4%	29	2.4%	28	1.5%	6 (8.82%)	<0.001
More than one time/month	5	6.8%	10	6.9%	74	6%	67	3.7%	22 (12.36%)	
One time/week	9	12.1%	13	9%	86	7%	103	5.6%	15 (6.64%)	
More than one time/week	26	35.1%	48	33.3%	362	29.4%	418	22.8%	64 (6.97%)	
One time/day	20	27%	46	31.9%	400	32.4%	731	39.8%	98 (7.57%)	
More than one time/day	11	14.9%	25	17.5%	282	22.8%	488	26.6%	44 (5.18%)	
Missing	13	14.94%	18	11.11%	61	4.71%	27	1.45%	189 (4.9%) **	557 (14.5%) ***
**Fresh fruit** **/Studies–Female**	**Primary**		**Middle School**		**High School**		**Higher Education**		**Missing**	*p* *
	**N.**	**%**	**N.**	**%**	**N.**	**%**	**N.**	**%**		
Never	1	1.5%	2	1.3%	29	2.8%	32	1.4%	4 (5.88%)	<0.001
More than one time/month	2	2.8%	9	5.9%	76	7.4%	88	4%	3 (1.69%)	
One time/week	6	8.3%	17	11.2%	73	7.1%	129	5.8%	1 (0.44%)	
More than one time/week	33	45.8%	41	27%	286	28%	540	24.2%	18 (1.96%)	
One time/day	14	19.4%	53	34.9%	324	31.7%	884	39.7%	20 (1.54%)	
More than one time/day	16	22.2%	30	19.7%	235	23%	554	24.9%	15 (1.76%)	
Missing	15	17.24%	24	13.64%	55	5.1%	43	1.89%	171 (4.4%) **	369 (9.6%) ***

* Fisher’s Exact Test, ** Missing data with none of the characteristics observed, *** Total missing.

**Table 3:** Multinomial logistic regression models used in predicting effects of parents’ studies across children’s status of food consumption.

Model		Univariable		Multivariable	
Dependent Variable = Candy Consumption		OR (95% C.I.)	*p* *	OR (95% C.I.)	*p* *
More than one time/month	Academic–Male (IV)	1.274 (1.021–1.589)	0.032	1.202 (0.906–1.595)	0.203
	Academic–Female (IV)	1.245 (0.995–1.557)	0.056	1.115 (0.830–1.499)	0.469
One time/week	Academic–Male (IV)	1.191 (0.919–1.544)	0.187	1.276 (0.914–1.783)	0.152
	Academic–Female (IV)	1.034 (0.797–1.340)	0.802	0.898 (0.635–1.270)	0.543
More than one time/week	Academic–Male (IV)	1.233 (0.968–1.569)	0.089	1.240 (0.909–1.691)	0.174
	Academic–Female (IV)	1.146 (0.898–1.463)	0.274	0.994 (0.719–1.373)	0.971
One time/day	Academic–Male (IV)	0.947 (0.692–1.296)	0.733	1.069 (0.711–1.605)	0.749
	Academic–Female (IV)	0.871 (0.638–1.188)	0.383	0.855 (0.562–1.299)	0.462
More than one time/day	Academic–Male (IV)	0.537 (0.354–0.813)	0.003	0.971 (0.563–1.675)	0.915
	Academic–Female (IV)	0.396 (0.265–0.591)	<0.001	0.401 (0.233–0.690)	0.001
Reference category: Never					
**Dependent variable = Soft drink consumption**		**OR (95% C.I.)**	*p* *	**OR (95% C.I.)**	*p* *
More than one time/month	Academic–Male (IV)	0.891 (0.698–1.138)	0.356	0.943 (0.696–1.278)	0.704
	Academic–Female (IV)	0.869 (0.670–1.128)	0.292	0.943 (0.674–1.318)	0.731
One time/week	Academic–Male (IV)	0.708 (0.542–0.925)	0.011	0.819 (0.587–1.142)	0.239
	Academic–Female (IV)	0.675 (0.509–0.895)	0.006	0.775 (0.539–1.114)	0.168
More than one time/week	Academic–Male (IV)	0.495 (0.382–0.642)	<0.001	0.652 (0.471–0.902)	0.010
	Academic–Female (IV)	0.472 (0.360–0.619)	<0.001	0.628 (0.442–0.891)	0.009
One time/day	Academic–Male (IV)	0.362 (0.262–0.499)	<0.001	0.696 (0.461–1.053)	0.086
	Academic–Female (IV)	0.266 (0.192–0.368)	<0.001	0.346 (0.225–0.530)	<0.001
More than one time/day	Academic–Male (IV)	0.199 (0.137–0.288)	<0.001	0.505 (0.315–0.809)	0.004
	Academic–Female (IV)	0.143 (0.100–0.206)	<0.001	0.231 (0.144–0.371)	<0.001
Reference category: Never					
**Dependent variable = Honey consumption**		**OR (95% C.I.)**	*p* *	**OR (95% C.I.)**	*p* *
More than one time/month	Academic–Male (IV)	1.309 (1.070–1.600)	0.009	1.044 (0.806–1.353)	0.742
	Academic–Female (IV)	1.551 (1.269–1.895)	<0.001	1.503 (1.151–1.963)	0.003
One time/week	Academic–Male (IV)	1.435 (1.145–1.798)	0.002	1.384 (1.033–1.855)	0.029
	Academic–Female (IV)	1.324 (1.060–1.654)	0.013	1.058 (0.785–1.428)	0.710
More than one time/week	Academic–Male (IV)	1.611 (1.280–2.027)	<0.001	1.392 (1.035–1.872)	0.029
	Academic–Female (IV)	1.604 (1.274–2.018)	<0.001	1.279 (0.942–1.738)	0.115
One time/day	Academic–Male (IV)	1.853 (1.369–2.508)	<0.001	1.582 (1.070–2.339)	0.021
	Academic–Female (IV)	1.739 (1.281–2.361)	<0.001	1.283 (0.853–1.930)	0.231
More than one time/day	Academic–Male (IV)	0.662 (0.346–1.265)	0.212	1.186 (0.505–2.785)	0.695
	Academic–Female (IV)	0.438 (0.236–0.814)	0.009	0.411 (0.175–0.966)	0.041
Reference category: Never					
**Dependent variable = Pastry consumption**		**OR (95% C.I.)**	*p* *	**OR (95% C.I.)**	*p* *
Never	Academic–Male (IV)	1.072 (0.616–1.867)	0.806	0.951 (0.453–1.994)	0.893
	Academic–Female (IV)	1.175 (0.672–2.056)	0.572	1.210 (0.571–2.566)	0.619
More than one time/month	Academic–Male (IV)	1.062 (0.780–1.448)	0.701	0.861 (0.571–1.297)	0.473
	Academic–Female (IV)	1.246 (0.921–1.687)	0.153	1.380 (0.911–2.090)	0.129
One time/week	Academic–Male (IV)	1.140 (0.853–1.523)	0.375	0.757 (0.517–1.108)	0.152
	Academic–Female (IV)	1.557 (1.169–2.074)	0.002	1.953 (1.322–2.884)	0.001
More than one time/week	Academic–Male (IV)	1.323 (1.018–1.719)	0.036	0.965 (0.681–1.368)	0.842
	Academic–Female (IV)	1.587 (1.227–2.053)	<0.001	1.633 (1.147–2.324)	0.006
One time/day	Academic–Male (IV)	1.347 (1.026–1.769)	0.032	0.981 (0.683–1.408)	0.918
	Academic–Female (IV)	1.596 (1.221–2.087)	0.001	1.656 (1.147–2.390)	0.007
Reference category: More than one time/day					
**Dependent variable = Fresh fruit consumption**		**OR (95% C.I.)**	*p* *	**OR (95% C.I.)**	*p* *
Never	Academic–Male (IV)	0.537 (0.319–0.902)	0.019	0.710 (0.355–1.422)	0.334
	Academic–Female (IV)	0.507 (0.304–0.845)	0.009	0.632 (0.316–1.265)	0.195
More than one time/month	Academic–Male (IV)	0.491 (0.347–0.694)	<0.001	0.597 (0.379–0.941)	0.026
	Academic–Female (IV)	0.513 (0.369–0.713)	<0.001	0.705 (0.448–1.112)	0.133
One time/week	Academic–Male (IV)	0.621 (0.458–0.843)	0.002	0.674 (0.454–0.999)	0.050
	Academic–Female (IV)	0.682 (0.505–0.921)	0.012	0.872 (0.583–1.305)	0.505
More than one time/week	Academic–Male (IV)	0.625 (0.514–0.759)	<0.001	0.618 (0.480–0.795)	<0.001
	Academic–Female (IV)	0.761 (0.625–0.926)	0.006	0.996 (0.767–1.294)	0.976
One time/day	Academic–Male (IV)	1.022 (0.851–1.227)	0.814	0.968 (0.763–1.227)	0.787
	Academic–Female (IV)	1.147 (0.952–1.382)	0.150	1.092 (0.851–1.402)	0.487
Reference category: More than one time/day					

IV = Independent variable, Non-academic parents = Reference group for IV, * Adjusted significance value to be significant for *p* < 0.01, Academic studies = higher education (ISCED 5–7), Non-academic studies = primary education (ISCED 1)/middle school education (ISCED 2)/high school education (ISCED 3).

**Table 4:** Social aspects of children according to parents’ studies.

Avoids Smiling /Studies–Male	Primary		Middle School		High School		Higher Education		Missing	*p* *
	N.	%	N.	%	N.	%	N.	%		
Negative	69	86.3%	134	90.5%	1136	92.4%	1728	95.9%	227 (6.89%)	<0.001
Affirmative	11	13.7%	14	9.5%	93	7.6%	74	4.1%	28 (12.73%)	
Missing	7	8.05%	14	8.64%	65	5.02%	60	3.22%	183 (4.7%) **	584 (15.2%) ***
**Avoids Smiling** **/Studies–Female**	**Primary**		**Middle School**		**High School**		**Higher Education**		**Missing**	*p* *
	**N.**	**%**	**N.**	**%**	**N.**	**%**	**N.**	**%**		
Negative	71	89.9%	141	87%	938	92.1%	2086	95.2%	58 (1.76%)	<0.001
Affirmative	8	10.1%	21	13%	80	7.9%	106	4.8%	5 (2.27%)	
Missing	8	9.2%	14	7.95%	60	5.57%	78	3.44%	169 (4.4% **)	392 (10.2%) ***
**Problems with** **other children** **/Studies–Male**	**Primary**		**Middle School**		**High School**		**Higher Education**		**Missing**	*p* *
	**N.**	**%**	**N.**	**%**	**N.**	**%**	**N.**	**%**		
Negative	69	93.2%	135	94.4%	1174	97.8%	1774	99.3%	242 (7.13%)	<0.001
Affirmative	5	6.8%	8	5.6%	27	2.2%	13	0.7%	10 (15.87%)	
Missing	13	14.94%	19	11.73%	93	7.19%	75	4.03%	186 (4.8%) **	638(16.6%) ***
**Problems with** **other children** **/Studies–Female**	**Primary**		**Middle School**		**High School**		**Higher Education**		**Missing**	*p* *
	**N.**	**%**	**N.**	**%**	**N.**	**%**	**N.**	**%**		
Negative	71	94.7%	151	94.4%	956	97.1%	2155	99.2%	61 (1.8%)	<0.001
Affirmative	4	5.3%	9	5.6%	29	2.9%	17	0.8%	4 (6.35%)	
Missing	12	13.8%	16	9.1%	93	8.63%	98	4.32%	167 (4.3% **)	451 (11.7%) ***
**Not satisfied** **with dental aspect** **/Studies–Male**	**Primary**		**Middle School**		**High School**		**Higher Education**		**Missing**	*p* *
	**N.**	**%**	**N.**	**%**	**N.**	**%**	**N.**	**%**		
Negative	33	52.4%	70	55.6%	668	63%	1190	72.4%	119 (5.72%)	<0.001
Affirmative	30	47.6%	56	44.4%	392	37%	453	27.6%	104 (10%)	
Missing	24	27.59%	36	22.22%	234	18.08%	219	11.76%	215 (5.6%) **	951 (14.5%) ***
**Not satisfied** **with dental aspect** **/Studies–Female**	**Primary**		**Middle School**		**High School**		**Higher Education**		**Missing**	*p* *
	**N.**	**%**	**N.**	**%**	**N.**	**%**	**N.**	**%**		
Negative	33	50.8%	63	49.2%	540	61.9%	1414	70.9%	30 (1.44%)	<0.001
Affirmative	32	49.2%	65	50.8%	332	38.1%	580	29.1%	26 (2.51%)	
Missing	22	25.3%	48	27.27%	206	19.11%	276	12.16%	176 (4.6%) **	784 (20.4%) ***
**Painful social problems** **/Studies–Male**	**Primary**		**Middle School**		**High School**		**Higher Education**		**Missing**	*p* *
	**N.**	**%**	**N.**	**%**	**N.**	**%**	**N.**	**%**		
Negative	66	80.5%	147	92.5%	1182	94%	1779	97.8%	250 (7.3%)	<0.001
Affirmative	16	19.5%	12	7.5%	76	6%	40	2.2%	13 (8.28%)	
Missing	5	5.75%	3	1.85%	36	2.78%	43	2.31%	175 (4.5% **)	525 (13.6%) ***
**Painful social problems** **/Studies–Female**	**Primary**		**Middle School**		**High School**		**Higher Education**		**Missing**	*p* *
	**N.**	**%**	**N.**	**%**	**N.**	**%**	**N.**	**%**		
Negative	68	82.9%	147	87%	963	92.7%	2180	98.1%	66 (1.93%)	<0.001
Affirmative	14	17.1%	22	13%	76	7.3%	43	1.9%	2 (1.27%)	
Missing	5	5.75%	7	3.98%	39	3.62%	47	2.07%	164 (4.2%) **	330 (8.6%) ***

* Fisher’s Exact Test, ** Missing data with none of the characteristics observed, *** Total missing.

**Table 5:** Binomial logistic regression models used in predicting effects of parents’ studies across children’s social aspects.

Model	Univariable		Multivariable	
Dependent Variable = Avoids Smilling	OR (95% C.I.)	*p*	OR (95% C.I.)	*p*
Academic–Male (IV)	0.486 (0.360–0.655)	<0.001	0.502 (0.344–0.733)	<0.001
Academic–Female (IV)	0.536 (0.406–0.707)	<0.001	0.953 (0.658–1.380)	0.798
**Dependent variable = Problems with other children**	**OR (95% C.I.)**	***p***	**OR (95% C.I.)**	***p***
Academic–Male (IV)	0.252 (0.134–0.474)	<0.001	0.428 (0.188–0.976)	0.044
Academic–Female (IV)	0.221 (0.125–0.390)	<0.001	0.422 (0.198–0.898)	0.025
**Dependent variable = Not satisfied with dental aspect**	**OR (95% C.I.)**	***p***	**OR (95% C.I.)**	***p***
Academic–Male (IV)	0.614 (0.525–0.719)	<0.001	0.717 (0.586–0.877)	0.001
Academic–Female (IV)	0.608 (0.520–0.711)	<0.001	0.773 (0.628–0.951)	0.015
**Dependent variable = Painful social problems**	**OR (95% C.I.)**	***p***	**OR (95% C.I.)**	***p***
Academic–Male (IV)	0.302 (0.208–0.437)	<0.001	0.701 (0.433–1.136)	0.149
Academic–Female (IV)	0.207 (0.145–0.297)	<0.001	0.271 (0.169–0.436)	<0.001

IV = Independent variable, Non-academic parents = Reference group for IV, Academic studies = higher education (ISCED 5–7), Non-academic studies = primary education (ISCED 1)/middle school education (ISCED 2)/high school education (ISCED 3).

**Table 6:** Medical aspects of children according to parents’ studies.

Tough Food Difficulty /Studies–Male	Primary		Middle School		High School		Higher Education		Missing	*p* *
	N.	%	N.	%	N.	%	N.	%		
Negative	57	67.9%	116	75.3%	1049	84.8%	1659	91.7%	207 (6.7%)	<0.001
Affirmative	27	32.1%	38	24.7%	188	15.2%	150	8.3%	62 (13.33%)	
Missing	3	3.45%	8	4.94%	57	4.4%	53	2.85%	169 (4.4%) **	559 (14.5%) ***
**Tough Food Difficulty** **/Studies–Female**	**Primary**		**Middle School**		**High School**		**Higher Education**		**Missing**	*p* *
	**N.**	**%**	**N.**	**%**	**N.**	**%**	**N.**	**%**		
Negative	52	62.7%	114	67.9%	857	83.2%	2004	91%	61 (1.98%)	<0.001
Affirmative	31	37.3%	54	32.1%	173	16.8%	188	9%	9 (1.94%)	
Missing	4	4.6%	8	4.55%	48	4.45%	68	3%	162 (4.2%) **	360(9.3%) ***
**Pain Frequency** **/Studies–Male**	**Primary**		**Middle School**		**High School**		**Higher Education**		**Missing**	*p* *
	**N.**	**%**	**N.**	**%**	**N.**	**%**	**N.**	**%**		
Never	19	22.1%	46	29.8%	336	26.5%	664	36.6%	64 (5.67%)	<0.001
Rarely	24	27.9%	52	33.5%	575	45.4%	753	41.4%	120 (7.87%)	
Occasional	25	29.1%	43	27.7%	306	24.2%	347	19.1%	99 (12.07%)	
Frequently	18	20.9%	14	9%	50	3.9%	52	2.9%	24 (15.19%)	
Missing	1	1.15%	7	4.32%	27	2.09%	46	2.47%	131 (3.4%) **	519 (13.5%) ***
**Pain Frequency** **/Studies–Female**	**Primary**		**Middle School**		**High School**		**Higher Education**		**Missing**	*p* *
	**N.**	**%**	**N.**	**%**	**N.**	**%**	**N.**	**%**		
Never	18	21.4%	34	19.7%	269	25.3%	788	35.6%	20 (1.77%)	<0.001
Rarely	25	29.8%	66	38.1%	476	45%	931	42%	26 (1.71%)	
Occasional	24	28.6%	51	29.5%	261	24.7%	433	19.6%	51 (6.22%)	
Frequently	17	20.2%	22	12.7%	51	4.8%	62	2.8%	6 (3.8%)	
Missing	3	3.45%	3	1.7%	21	1.95%	56	2.47%	129 (3.3%) **	315 (8.2%) ***

* Fisher’s Exact Test, ** Missing data with none of the characteristics observed, *** Total missing.

**Table 7:** Multinomial and binomial logistic regression models used in predicting effects of parents’ studies across children’s medical aspects.

Model		Univariable		Multivariable	
**Dependent variable = Tough Food Difficulty**		**OR (95% C.I.)**	***p***	**OR (95% C.I.)**	***p***
Academic–Male (IV)		0.437 (0.352–0.541)	<0.001	0.655 (0.495–0.868)	0.003
Academic–Female (IV)		0.392 (0.321–0.478)	<0.001	0.533 (0.405–0.701)	<0.001
**Dependent variable = Chewing Difficulty**		**OR (95% C.I.)**	***p***	**OR (95% C.I.)**	***p***
Academic–Male (IV)		0.583 (0.359–0.948)	0.030	1.162 (0.601–2.248)	0.655
Academic–Female (IV)		0.376 (0.234–0.604)	<0.001	0.325 (0.168–0.631)	0.001
**Dependent variable–Pain Frequency**		**OR (95% C.I.)**	*p* *	**OR (95% C.I.)**	*p* *
Rarely	Academic–Male (IV)	0.699 (0.594–0.822)	<0.001	0.826 (0.670–1.019)	0.075
	Academic–Female (IV)	0.669 (0.566–0.790)	<0.001	0.744 (0.596–0.930)	0.009
Occasional	Academic–Male (IV)	0.560 (0.463–0.679)	<0.001	0.755 (0.587–0.969)	0.028
	Academic–Female (IV)	0.525 (0.433–0.637)	<0.001	0.602 (0.465–0.781)	<0.001
Frequently	Academic–Male (IV)	0.383 (0.265–0.554)	<0.001	0.732 (0.447–1.198)	0.215
	Academic–Female (IV)	0.281 (0.198–0.398)	<0.001	0.347 (0.212–0.566)	<0.001
Reference category: Never					
**Dependent variable–Reasons for medical visit**		**OR (95% C.I.)**	*p* **	**OR (95% C.I.)**	*p* **
Treatment	Academic–Male (IV)	0.723 (0.337–1.551)	0.405	-	-
	Academic–Female (IV)	0.554 (0.239–1.282)	0.167	-	-
Pain	Academic–Male (IV)	0.549 (0.281–1.071)	0.078	-	-
	Academic–Female (IV)	0.242 (0.117–0.498)	<0.001	-	-
Reference category: Routine check					

IV = Independent variable, Non-academic parents = Reference group for IV, * Adjusted significance value to be significant for *p* < 0.01, ** Adjusted significance value to be significant for *p* < 0.0166, Academic studies = higher education (ISCED 5–7), Non-academic studies = primary education (ISCED 1)/middle school education (ISCED 2)/high school education (ISCED 3).

## Data Availability

The data presented in this study are available from the corresponding authors upon reasonable request.

## References

[1] Glick M., Williams D.M., Kleinman D.V., Vujicic M., Watt R.G., Weyant R.J. (2017). A new definition for oral health developed by the FDI World Dental Federation opens the door to a universal definition of oral health. Am. J. Orthod. Dentofacial Orthop..

[2] Hescot P. (2017). The New Definition of Oral Health and Relationship between Oral Health and Quality of Life. Chin. J. Dent. Res..

[3] Ahonen H., Pakpour A., Norderyd O., Broström A., Fransson E.I., Lindmark U. (2022). Applying World Dental Federation Theoretical Framework for Oral Health in a General Population. Int. Dent. J..

[4] Campos L.A., Peltomäki T., Marôco J., Campos J.A.D.B. (2021). Use of Oral Health Impact Profile-14 (OHIP-14) in Different Contexts. What Is Being Measured?. Int. J. Environ. Res. Public Health.

[5] Smith G.D., Neaton J.D., Wentworth D., Stamler R., Stamler J. (1996). Socioeconomic differentials in mortality risk among men screened for the Multiple Risk Factor Intervention Trial: I. White men. Am. J. Public. Health.

[6] Cunha M.A., Vettore M.V., Santos T.R.D., Matta-Machado A.T., Lucas S.D., Abreu M.H.N.G. (2020). The Role of Organizational Factors and Human Resources in the Provision of Dental Prosthesis in Primary Dental Care in Brazil. Int. J. Environ. Res. Public Health.

[7] Shaharyar S.A., Bernabé E., Delgado-Angulo E.K. (2021). The Intersections of Ethnicity, Nativity Status and Socioeconomic Position in Relation to Periodontal Status: A Cross-Sectional Study in London, England. Int. J. Environ. Res. Public Health.

[8] World Health Organization Global Oral Health Status Report: Towards Universal Health Coverage for Oral Health by 2030: Executive Summary. https://www.who.int/publications/i/item/9789240061569.

[9] Palmer C. (2008). Important Relationships Between Diet, Nutrition, and Oral Health. Nutr. Clin. Care.

[10] Moynihan P. (2002). Dietary advice in dental practice. Br. Dent. J..

[11] Najeeb S., Zafar M.S., Khurshid Z., Zohaib S., Almas K. (2016). The Role of Nutrition in Periodontal Health: An Update. Nutrients.

[12] Salas M.M., Nascimento G.G., Vargas-Ferreira F., Tarquinio S.B., Huysmans M.C., Demarco F.F. (2015). Diet influenced tooth erosion prevalence in children and adolescents: Results of a meta-analysis and meta-regression. J. Dent..

[13] Lieffers J.R.L., Vanzan A.G.T., Rover de Mello J., Cammer A. (2021). Nutrition Care Practices of Dietitians and Oral Health Professionals for Oral Health Conditions: A Scoping Review. Nutrients.

[14] Pflipsen M., Zenchenko Y. (2017). Nutrition for oral health and oral manifestations of poor nutrition and unhealthy habits. Gen. Dent..

[15] American Academy on Pediatric Dentistry Clinical Affairs Committee, American Academy on Pediatric Dentistry Council on Clinical Affairs (2008). Policy on dietary recommendations for infants, children, and adolescents. Pediatr. Dent..

[16] Health Scotland Oral Health and Nutrition Guidance for Professionals. Quick Reference Guide. https://www.healthscotland.scot/media/2338/oral-health-and-nutrition-quick-guide.pdf.

[17] American Dental Association Nutrition and Oral Health. https://www.ada.org/resources/research/science-and-research-institute/oral-health-topics/nutrition-and-oral-health.

[18] Bulgareli J.V., Faria E.T., Cortellazzi K.L., Guerra L.M., Meneghim M.C., Ambrosano G.M.B., Frias A.C., Pereira A.C. (2018). Factors influencing the impact of oral health on the daily activities of adolescents, adults and older adults. Rev. Saude Publica.

[19] Peres K.G., Cascaes A.M., Leão A.T., Côrtes M.I., Vettore M.V. (2013). Aspectos sociodemográficos e clínicos da qualidade de vida relacionada à saúde bucal em adolescentes [Sociodemographic and clinical aspects of quality of life related to oral health in adolescents]. Rev. Saude Publica.

[20] Mariño R., Glenister K., Bourke L., Morgan M., Atala-Acevedo C., Simmons D. (2021). Patterns of use of oral healthcare services in rural adults: The Crossroads II Dental sub-study. Aust. Dent. J..

[21] Kaur M. (2013). School Children Knowledge Regarding Dental Hygiene. IOSR J. Nurs. Health Sci..

[22] Abu-Elenen N.R., Abdella N.H., Elkazaz R.H. (2015). Effect of an Oral Care Educational Program on the Knowledge, Practice and Self-Efficacy Among School Age Children. Int. J. Res. Stud. Biosci..

[23] National Institute of Public Health https://insp.gov.ro/download/cnepss/stare-de-sanatate/boli_netransmisibile/sanatate_orala/Analiza-de-situatie-2022.pdf.

[24] National Institute of Public Health https://insp.gov.ro/wpfb-file/metodologie-sanatate-orala-revizuita-19-aprilie-2022-pdf/.

[25] Eurostat Statistic Explained. https://ec.europa.eu/eurostat/statistics-explained/index.php?title=International_Standard_Classification_of_Education_(ISCED)#Implementation_of_ISCED_2011_.28levels_of_education.29.

[26] Perpelea A.-C., Sfeatcu R., Tănase M., Meleșcanu Imre M., Ripszky Totan A., Cernega A., Funieru C., Pițuru S.-M. (2024). A STEPwise Approach for Oral Hygiene Behavior of Schoolchildren in Romania. Healthcare.

[27] Tellez M., Zini A., Estupiñan-Day S. (2014). Social Determinants and Oral Health: An Update. Curr. Oral. Health Rep..

[28] de Abreu M.H.N.G., Cruz A.J.S., Borges-Oliveira A.C., Martins R.d.C., Mattos F.d.F. (2021). Perspectives on Social and Environmental Determinants of Oral Health. Int. J. Environ. Res. Public Health.

[29] Akhter R. (2019). Epidemiology of Temporomandibular Disorder in the General Population: A Systematic Review. Adv. Dent. Oral. Health.

[30] Balgiu B.A., Sfeatcu R., Mihai C., Lupușoru M., Bucur M.V., Tribus L. (2022). Romanian Version of the Oral Health Values Scale: Adaptation and Validation. Medicina.

[31] Vyas H., Patel K.K., Chaudhary B., Jani K. (2021). Impact of socio-economic factors on oral health. Int. J. Creat. Res. Thoughts.

[32] Wright F. (2015). Social implications and workforce issues in the oral health of an ageing population. Aust. Dent. J..

[33] Baum F. (2019). The Power of Policy to Promote Health and Well-Being’, Governing for Health: Advancing Health and Equity through Policy and Advocacy.

[34] Ricciardi W., Ricciardi W., Souza L., Souza L., Amofah G., Amofah G., Macartney K., Macartney K., Lomazzi M., Lomazzi M. (2020). 2.I. Workshop: Implementing local health equity policies in Europe—Needs, governance and tools. Eur. J. Public Health.

[35] Kuhn J., Trojan A. (2010). Gesundheit fördern statt kontrollieren—Lessons learned, lessons to learn [Promoting instead of controlling health--lessons learned, lessons to learn]. Gesundheitswesen.

[36] Ramirez-Rubio O., Daher C., Fanjul G., Gascon M., Mueller N., Pajín L., Plasencia A., Rojas-Rueda D., Thondoo M., Nieuwenhuijsen M.J. (2019). Urban health: An example of a “health in all policies” approach in the context of SDGs implementation. Global Health.

[37] Richter K.D., Acker J., Scholz F., Niklewski G. (2010). Health promotion and work: Prevention of shift work disorders in companies. EPMA J..

[38] Koivusalo M. (2010). The state of Health in All policies (HiAP) in the European Union: Potential and pitfalls. J. Epidemiol. Community Health.

[39] Greer S.L., Jarman H. (2021). What Is EU Public Health and Why? Explaining the Scope and Organization of Public Health in the European Union. J. Health Polit. Policy Law.

[40] Martin-Olmedo P., O’Mullane M., EUPHA-HIA, EUPHA-PHPP, EUPHA-ECO, EUPHA-LAW (2022). 9.G. Workshop: Health in all Policies: Key driver for better health still awaiting of greater governing stewardship. Eur. J. Public. Health.

[41] Berkman L.F., Kawachi I., Glymour M. (2014). Social Epidemiology.

[42] Petersen P.E. (2008). World Health Organization global policy for improvement of oral health—World Health Assembly 2007. Int. Dent. J..

[43] Detels R., Karim Q.A., Baum F., Li L., Leyland A.H. (2021). Oxford Textbook of Global Public Health.

[44] Barten F., Mitlin D., Mulholland C., Hardoy A., Stern R. (2007). Integrated approaches to address the social determinants of health for reducing health inequity. J. Urban. Health.

[45] Picard M., Sabiston C.M., McNamara J.K. (2011). The need for a trans-disciplinary, global health framework. J. Altern. Complement. Med..

[46] Dourado Martins J., Oliveira Mascarenhas Andrade J., Souza Freitas V., de Araújo T.M. (2014). Determinantes sociais de saúde e a ocorrência de câncer oral: Uma revisão sistemática de literatura [Social determinants of health and the occurrence of oral cancer: A systematic literature review]. Rev. Salud. Publica.

[47] Gomaa N., Glogauer M., Tenenbaum H., Siddiqi A., Quiñonez C. (2016). Social-Biological Interactions in Oral Disease: A ‘Cells to Society’ View. PLoS ONE.

[48] Newton J.T., Bower E.J. (2005). The social determinants of oral health: New approaches to conceptualizing and researching complex causal networks. Community Dent. Oral. Epidemiol..

[49] Geetha Priya P.R., Asokan S., Janani R.G., Kandaswamy D. (2019). Effectiveness of school dental health education on the oral health status and knowledge of children: A systematic review. Indian. J. Dent. Res..

[50] Parliament of Romania. Law nr. 198 of 4 July 2023, Pre-University Education. https://legislatie.just.ro/Public/DetaliiDocumentAfis/271896.

[51] Sawyer S.M., Azzopardi P.S., Wickremarathne D., Patton G.C. (2018). The age of adolescence. Lancet Child. Adolesc. Health.

[52] Aleksejūnienė J., Pūrienė A., Rimkervicius A., Amariei C., Oancea R., Porosencova T., Porosencov E., Nikolovska J., Mirnaya E., Serova-Papakul A. (2020). Knowledge, dentist confidence and management of periodontal patients among general dentists from Belarus, Lithuania, Macedonia, Moldova and Romania. BMC Oral. Health.

